# A dyadic planning intervention to quit smoking in single-smoking couples: design of a randomized controlled trial

**DOI:** 10.1186/s40359-018-0266-8

**Published:** 2018-11-12

**Authors:** Anne H. Buitenhuis, Marrit A. Tuinman, Mariët Hagedoorn

**Affiliations:** Department of Health Psychology, University Medical Center Groningen, University of Groningen, De Brug, FA12, POB 30.001, 9700 RB Groningen, The Netherlands

**Keywords:** Implementation intentions, Smoking cessation, Tobacco, Dyadic, Relationship satisfaction, Couple, Non-smoking partner, Diary, Ecological momentary assessment, Randomized controlled trial

## Abstract

**Background:**

Tobacco use is the largest preventable cause of death. Smoking cessation interventions that use implementation intentions show promising results. Implementation intentions are if-then plans that specify a certain behaviour within a situational context. This study will examine whether involving a non-smoking partner could improve planning interventions, and whether and which partner interactions underlie this effectiveness.

**Methods:**

This single-blind randomized controlled trial has a longitudinal design with a baseline questionnaire, end-of-day measurements for three weeks starting on the quit date, and a follow-up questionnaire after three months.

Participants: single-smoking couples who live together and are in a relationship for more than one year.

Setting: couples are randomized to either a dyadic or individual planning condition. After the intervention the smoker attempts to quit smoking and the diary measurements start.

Measurements: The primary outcome variable is smoking abstinence. Secondary outcome measures are smoking behaviour and relationship satisfaction. Partner interactions are examined as a possible mediator.

**Discussion:**

This RCT is the first to examine the effectiveness of dyadic planning to quit smoking in single-smoking couples. Partner interactions are thought to play an important role during the quit attempt, and therefore in the effectiveness of the intervention. This RCT will provide more insight into which daily partner interactions are beneficial for smoking abstinence and the couples’ relationship satisfaction, and whether the type of intervention is related to different types or levels of partner interactions and smoking behaviour. When proven effective, this planning intervention in combination with coaching for the non-smoking partner will be a valuable and low-cost addition to existing smoking interventions.

**Trial registration:**

The trial is retrospectively registered on 19/04/2017 on www.trialregister.nl (TC: 6398).

**Electronic supplementary material:**

The online version of this article (10.1186/s40359-018-0266-8) contains supplementary material, which is available to authorized users.

## Background

Despite discouraging measures and intervention programs to help individuals to quit smoking, the percentage of smokers in the Netherlands is still about 24% [1]. With tobacco smoking as the largest preventable cause of death [2], successful smoking cessation interventions are important. The use of implementation intentions seems a promising method to help smokers to quit [3]. This RCT examines whether involving a non-smoking partner could improve planning interventions, and whether and which partner interactions underlie this effectiveness.

Implementation intentions are if-then plans that specify a certain behaviour within a situational context [4]. These intentions show enduring changes in behaviour and therefore increase the likelihood to achieve one’s goals. An example of an implementation intention is: “After dinner when I crave a cigarette, I’m going for a walk instead of smoking a cigarette.” Planning interventions (in which implementation intentions are formed) are in general effective in changing health behaviour [5], and it shows promising results for smoking behaviour [3]. Specifically, forming if-then plans (e.g., of what to do when one has cravings or when one is in a situation where one usually smokes) has been found to decrease smoking habits (i.e. smoking automatically without thinking about it) and the number of cigarettes smoked in a group of current smokers.

To enhance the efficacy of implementation intention interventions, involving the spouse could be beneficial [6]. The health behaviour of a spouse is an important influencing factor of one’s own health behaviour, including smoking [7]. Research shows that smokers partnered with a non-smoker (i.e. single-smoking couples), compared to those partnered with a smoker, more often try to quit [8, 9], use less tobacco [10] and have an increased likelihood of (successful) smoking cessation [11]. Approximately 35% of the smokers have a non-smoking partner [11, 12]. Additionally, non-smokers, compared to smokers, are more willing to support their partner’s quit attempt and to use influence tactics such as complimenting on not smoking or criticizing smoking behaviour [13]. This intended support is even higher when partners are more satisfied with their relationship*.* Receiving partner support seems an important contributing factor to successful smoking cessation. When smokers bring a support partner to a smoking cessation therapy, the abstinence rate is significantly greater and more likely to be sustained for at least one month [14]. Support from a non-smoking partner, both prior to and during a self-set quit attempt, was related to less smoking [15]. After the quit date, when support is needed the most, changes in support were even stronger related to changes in number of cigarettes smoked. All in all, one could say that smokers who are in a single-smoking relationship are in a favourable social environment to quit smoking. The next step is to examine how this favourable environment can be used to help the smoker to quit.

In our RCT, we aim to involve the non-smoking partner before the quit date by asking the couple to jointly create an if-then plan for the smoker to quit smoking. This would change the role of the partner from just a support provider to a part of the team: quitting smoking becomes a dyadic effort. In dyadic planning, the partner can help by thinking of and supporting plans such as how to resist the temptation of smoking a cigarette. We will examine the effect of dyadic planning to quit smoking in comparison to individual planning, where the smokers make a plan on their own.

Research on dyadic planning is limited. To the best of our knowledge, there are no studies focusing on dyadic planning to quit smoking. Dyadic planning has been studied in the context of implementing a new (health) behaviour. However, it did not help to involve the partner in planning to increase daily physical activity in target persons and their partners [16], nor to integrate pelvic-floor exercise in daily life after prostatectomy [17]. On the other hand, dyadic planning (compared to individual planning) was more successful regarding maintenance of these exercises [18]. Therefore, dyadic planning might be a promising method to prevent relapse. Dyadic planning also seemed to buffer the detrimental effects that negative control of the partner had on behaviour change [17]. Perhaps the controlling behaviour of the partner feels justified because the couple is operating as a team; social control might be perceived differently.

Several aspects might explain why no intervention effects were found. As the authors state, dyadic planning might occur in all couples as a general adjustment strategy after surgery [17], so partners could have involved themselves in the planning in both conditions. Dyadic planning failed to increase physical activity in target persons and their partners in daily life [16]. Although this study had a target person that was the focus in the planning, this target person was randomly chosen within the couple and the plans could apply to both partners. Accordingly, increased activity was expected in both partners: the partners’ activity was also monitored. This might compromise the measure of the effect of the dyadic planning, since when the partner is not changing his or her behaviour, the target person might be less motivated to do so. In our study, the drafted plans are only applicable to the smoker, therefore creating a different situation within the couple. The target person is clear, and the role of the partner is to help with the plan and to support the smoker.

Collaborative planning has also been studied in the context of implementing a new (health) behaviour and results seem promising. It can be distinguished from dyadic planning, in that the partner is not only involved in creating the plan, but also plays a role in the plan itself (e.g., enacting a behaviour together) [6]. When the partner was involved in creating collaborative implementation intentions to increase physical activity in counsel employees, it was more effective [19]. These participants lost more weight over time and were more physically active then participants who made intentions individually. Since, smoking in single-smoking couples is likely an individual activity, often performed in the absence of the partner, plans have to be created that can be acted upon alone. However, couples are allowed to integrate collaborative plans for times they are together (e.g. taking a walk together or playing a game when the smoker has a difficult moment in the evening). Therefore, the main elements of the intervention lie in the dyadic planning, while collaborative planning may be part of the plan only when the spouses make it so. We therefore consider this a dyadic planning intervention.

**Fig. 1:**
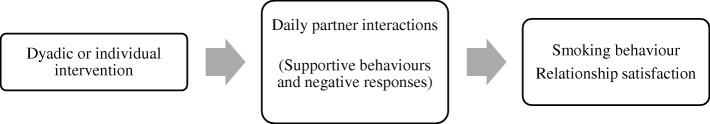
The proposed model with partner interactions as a mediator

Measuring partner support is an important step to fully understand the effectiveness of and working mechanisms behind a dyadic and individual planning intervention. Therefore, in our RCT partner support that occurs naturally in both conditions will be examined (Fig. 1). The nature of support for the smoker does matter. A greater frequency of supportive behaviours of the partner predicts cessation attempts [20], while negative behaviours such as criticizing, nagging and policing in relation to smoking are impediments to smoking cessation [21]. The nature of support can fluctuate during the course of a quit attempt. During moments of relapse, the likelihood of negative support increases and the smoker is perceived as less committed to quitting smoking by their partner [22]. Also, quitting increases anger, irritability and frustration in smokers [23], which might trigger conflicts between spouses. Dyadic planning might act as a buffer for negative behaviours [17]. When the non-smoking partner is involved in creating implementation intentions, quitting smoking becomes more a dyadic effort than an individual effort. Support might be different or perceived as different when the couple is operating as a team, compared to when one partner of the couple is working on the task alone. This might explain why dyadic planning can act as a buffer for negative behaviours while individual planning does not show this effect [17]*.* So, dyadic planning could be more effective for successful smoking cessation, because it influences the provided support and behaviour of the partner, or the perception of these responses, or both.

### Aims of the present study

The primary aim of this study is to assess immediate effectiveness and effectiveness over time of a dyadic planning intervention compared to an individual planning intervention for smoking cessation in smokers with a non-smoking partner. We hypothesize that participants in the dyadic planning condition will report fewer cigarettes smoked and a longer duration of abstinence than participants in the individual planning condition. We also hypothesize that participants in the dyadic planning condition will report higher relationship satisfaction than participants in the individual condition.

The secondary aim of this study is to examine possible underlying mechanisms in effectiveness in terms of partner interactions. We will examine differences between the conditions in the nature and frequency of partner interactions and how they are related to daily smoking behaviour and relationship satisfaction. We hypothesize that partners in the dyadic condition show more supportive behaviours in general and after a weak moment (of relapse), more often discuss weak moments, the plan, and the quit attempt. Additionally, we hypothesize that negative responses of the partner in the dyadic condition are less strongly related to a decrease in relationship satisfaction than in the individual condition.

## Methods

### Participants

Participating couples must consist of one smoker (smoking cigarettes every day or multiple days per week) and one non-smoker, who are in a relationship for at least one year and living together. Both partners should have their own mobile phone, for receiving the daily questionnaire. Exclusion criteria are pregnancy and aged younger than 18. Participants from the Netherlands can enroll from April 2017 to July 2018.

### Procedure

**Fig. 2:**
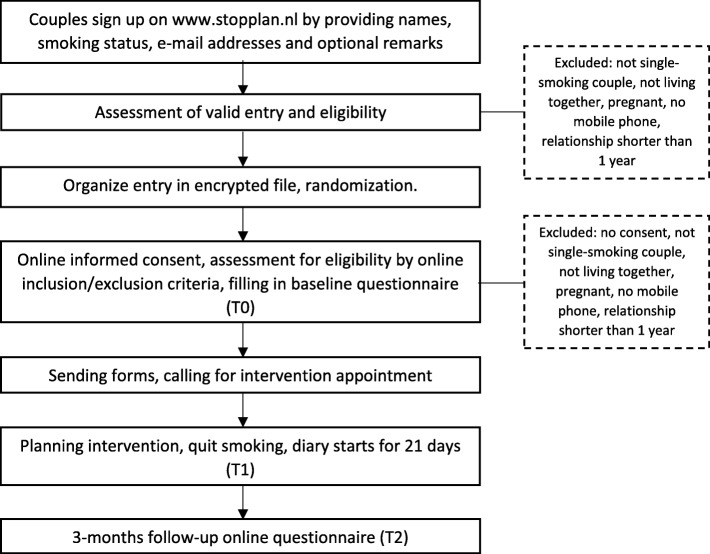
Participant timeline

This single-blind randomized controlled trial has a longitudinal design with a baseline questionnaire, measurements once a day for three weeks, and a follow-up questionnaire after three months (Fig. 2). Participants are recruited using social media (e.g., Facebook, Twitter), by repeatedly posting a short advertisement with a link to the website [24]. Facebook appears to be an effective channel to recruit single-smoking couples [25]. When couples want to participate, they can fill in an application form on the website, which asks for both members’ e-mail addresses and their smoking status. The applications are organized in an encrypted file, where they are randomly assigned to either the individual or dyadic condition, and receive a respondent number and couple code. Participants can stop participating at any time upon request.

#### Baseline (T0)

A message will be sent to the e-mail addresses participants provided through the website. This e-mail contains information about every part of the study, participation and privacy regulations. Every participant will get a personalized link to the baseline questionnaire (T0), where participants give their informed consent. At the end of this survey, participants will be asked to fill in their home address and phone number. These data will be added to the encrypted file with participant information. Then, a package will be sent to the given address, containing an information letter, an instruction manual with frequently asked questions (FAQs) about the diary period, and a closed colored envelope. This envelope contains either a blank individual or a dyadic planning sheet.

#### Intervention and diary period (T1)

A few days after sending this package, the intervention is offered by phone (more information below). After the intervention, participants are guided by the researcher to register their phone numbers on the website of the text message service (www.surveysignal.com). Participants get a few example questions, to practice filling in the diary, followed by instructions about the diary questionnaires. The day after filling in the planning sheet, the first text message will be sent by Survey signal. Every evening, for the following 21 days both partners get a text message with a link to the diary questionnaire. After 11 days, participants receive a motivating email, which states that they filled in half or more than half of the questionnaires and that we value their participation. When several diaries are missed, an email is send to ask if there are problems or if we can do anything to make their participation easier (e.g., changing the time of the text message). After completing the diary, participants receive a voucher of 20 euros as a compensation for their time.

#### Follow-up (T2)

Three months after the diary has ended, couples receive an email with a follow-up questionnaire (T2). Reminders will be send to increase the response rate. When both partner fill in this questionnaire, they will receive a second compensation, again a voucher of 20 euros. At the end of the questionnaire, participants will be debriefed and given information on how to receive the study results.

### Randomization

Couples are randomized by the researchers by means of flipping a coin. Conditions are predetermined (before registration of the couple) by this method for every block of ten couples, to exclude any kind of influence the researchers might have. The randomization is single-blind; couples do not know that there are two different conditions until the debriefing that is given after the follow-up questionnaire. When participants do not fill in the follow-up questionnaire, the debriefing is sent to them by e-mail.

### Detailed description of intervention and groups

The intervention is based on a planning sheet originally used to enhance pelvic floor exercise after prostatectomy among prostate cancer survivors [17]. Participants were asked when and where they were going to perform these exercises. Because quitting smoking is a different kind of behaviour, the sheet was adapted. Instead of focusing on starting with a health enhancing behaviour, it is focused on stopping an unhealthy behaviour. Text and examples were adapted to fit the situation of a smoker and we ask participants to sign the plan to make it more official. The main idea, of creating implementation intentions, remained the same.

### Condition 1: Dyadic planning intervention

The couple is asked to open the colored envelop and read the instructions out loud, so the researcher on the phone is also able to hear in which step the couple is. The first step is to write down moments that the smoker usually smokes. The partner is invited to help and also think of moments that might have been forgotten. If the partner does not involve him or herself in the task, the researcher will ask if he or she has any additions. The researcher makes sure that the list of moments is complete, for example by asking when the first and last cigarette on a day is smoked and whether there are any important moments that occur less frequently (e.g., birthdays, going out). The second step is writing down where these moments occur, and how to prevent smoking at these moments. Again, partners are asked to help the smoker. Plans have to be concrete actions; this is monitored by the researcher. For every moment a plan is made (e.g., going for a walk instead of smoking after lunch). Finally, the couple indicates how feasible each plan is on a scale from 1 to 5. On the last page of the planning sheets, the smoker writes down the quit date (the next day), and both partners put their signature to make it ‘official’. They are asked to take a picture of the plan and email it to us, when possible, during the phone call.

### Condition 2: Individual planning intervention

When the non-smoking partner is next to the smoker, the researcher asks him or her to leave the room and not be present while filling in the planning sheet. Then, the smoker is asked to open the colored envelope, and read the instructions out loud. The planning sheet and steps are similar to the dyadic planning condition, the only difference being the partners’ absence during the intervention. Therefore, only the smoker makes and signs the plan. When the smoker has sent the plan by email, the partner can reenter the room for the diary instructions.

### Measures

Table 1 shows all variables that are measured (including exploratory/potential covariates).

**Table 1:** Content of the baseline, diary and follow-up questionnaire

Measures	Baseline		Diary		Follow-up	
	*Smoker*	*Partner*	*Smoker*	*Partner*	*Smoker*	*Partner*
Baseline information						
Socio-demographics	X	X				
Smoking details and history	X	X			X	
Quit attempt history	X					
Primary & Secondary outcomes						
Number of cigarettes smoked	X		X	X	X	X
Hiding of smoking	X				X	
Duration of abstinence					X	X
Perceived frequency of smoking-related conflicts (SRC)	X	X			X	X
Relationship satisfaction	X	X	X	X	X	X
Smoking dependence	X		X	X	X	
Smoking now compared to before quit attempt					X	X
Mediators						
Details of partner contact			X	X		
Partner responses			X	X		
Partner responses to difficult moment			X	X		
Exploratory outcomes and potential covariates						
Attitude towards smoking	X	X				
Affect (PANAS)	X	X	X	X		
Opinion partner about quitting/smoking behaviour	X	X				X
Influence of SRC	X					
Perceived susceptibility	X	X			X	X
Perceived relatedness	X	X			X	X
Self-compassion towards relapse			X	X	X	X
Intention to quit	X				X	
Taking steps to go smoking			X			
Use of smoking replacement			X	X	X	X
Difficulty quitting			X	X		
Adherence to planning			X	X	X	X
Helpfulness of planning			X	X	X	X
Conflicts not related to smoking			X	X		
Interference of quitting with relationship			X	X		
Details social contacts			X	X		
Interference of quitting with social contact			X			
Other health behaviours	X	X				
SRC initiator	X	X				
SRC recentness	X	X				
SRC pattern	X	X				
Partner interaction questionnaire	X	X				
Details most difficult moment			X	X		
Distraction from smoking			X	X		
Motivation to remain quit			X			
Evaluation of diary			X	X	X	X
Self-efficacy to quit	X		X	X		
Daily activities			X	X		
Role of smoking in break-up					X	X
Influence of plan on relationship					X	X
Missing smoking					X	

#### Primary outcomes

The primary outcome is self-reported smoking abstinence from tobacco smoking. Every day, participants are asked whether they smoked and if so, how many cigarettes (1, 2, 3, 4, 5, > 5). In the follow-up questionnaire, participants are asked about their smoking behaviour to assess effectiveness over time.

#### Secondary outcomes

In the baseline and follow-up questionnaire, participants fill in the Dutch version of the Maudsley Marital Questionnaire [26, 27]. The scale contains 15 questions that are answered on 9-point Likert scales (e.g., ‘How much are you committed to this marriage?). Daily relationship satisfaction is measured by asking participants how satisfied they are with their relationship at that moment. The scale ranges from 1 = ‘Unhappy’ to 10 = ‘Very happy’.

#### Mediators

Daily partner interactions (supportive behaviours and negative responses) will be examined as a possible mediator in the relationship between the intervention and the primary outcomes. First of all, participants are asked whether they had contact with their partner today (e.g., talking, texting). If they had contact, they are asked how satisfied they are about this (1 = ‘Not at all’ to 7 = ‘Very much’). Details are asked about the time spent with the partner (e.g., how much time, when during the day). Supportive behaviours and negative responses are measured by seven questions about interactions about smoking and the planning (e.g., today my partner motivated me to remain abstinent, today my partner did not show interest in my planning, today my partner and I had a fight about my planning). The answer scale ranges from 1 = ‘Not at all’ to 7 = ‘Very much’. Participants are asked whether they discussed the most difficult moment of that day with their partner, and if so how he/she responded. Partner responses (supportive and negative) to difficult moments are measured by four items (i.e., supportive, motivating, angry/irritated, blaming) and participants are asked to score these on a scale from 1 = ‘Not at all’ to 7 = ‘Very much’.

### Data management

Online data is collected on the secured and password protected server of Unipark [28] and regular (anonymous) back-ups are saved on the secured and password protected server of the University Medical Center Groningen, only assessable by the involved researchers. The surveys are online and programmed in such a way that no questions can be skipped or answered different than the scale, therefore, range checks are not necessary. Data will be monitored for missing days during the study period.

### Statistical analyses

For our primary aim we will compare the proportion of successful abstainers in both groups, weekly (day 7, 14, 21) and after 3 months (primary outcome variable). Primary analyses are conducted with baseline and follow-up data according to the intention-to-treat approach. Secondly, we will compare the number of cigarettes smoked between the groups, controlling for baseline smoking (secondary outcome variable). Similar comparisons will be made for relationship satisfaction (secondary outcome variable). Additional independent and paired post-hoc t-tests will be performed to examine between-group and within-group differences, and chi-square tests for categorical variables*.* For the secondary aim, we will examine the effects of partner interactions on same and next-day smoking behaviour and relationship satisfaction (concurrent and lagged effects). Multilevel modelling will be used to analyse the diary data [29]. The diary data is nested within individuals and couples. Multi-level analyses will take into account between-person variation (individual variation from the grand mean) as well as within-person variation (an individual’s daily variation from their personal mean). To examine the indirect effect of the intervention on smoking and relationship satisfaction via partner interactions, we will conduct a within-person mediation analysis, allowing for random intercepts and slopes [29]. We will check whether the randomization was successful regarding demographical, smoking and relationship variables. If not, those variables will be taken into account as covariates in the analyses. 

### Power analysis

In literature, 15.48% of the participants successfully quit smoking after an intervention using implementation intentions, measured one month later [3]. Therefore, we expect an abstinence rate of 15% in the individual planning (control) group. With a power of 0.8 and an alpha of .05, we need 70 couples per group to detect a relevant increase in effectiveness of 20% (primary aim). Additionally, we consider a minimal decrease of 50% in number of cigarettes, between baseline and follow-up, relevant [30].

A minimum sample of 50 cases is advised in a multi-level design [31] and diary studies in this research area are usually between 60 and 100 participants. Considering a power of .80 and an alpha of .05, the power analysis showed that a net sample size of 82 couples is sufficient to find an effect size of at least .3 (a medium effect) in the diary between smoking-related interactions and smoking behaviour (secondary aim). Therefore, with the sample of 140 couples we have sufficient power to detect a relevant difference in effectiveness of the intervention as well as the research questions related to the diary data. To take into account drop-out, we aim to include 15% extra couples.

## Discussion

Our study is the first to examine the immediate effectiveness and effectiveness over time, of dyadic planning to quit smoking in single-smoking couples. Current research on dyadic planning is limited and shows mixed results. Previous RCTs mainly focused on the implementation of new behaviours (e.g., increasing activity or doing pelvic-floor exercises). This RCT will give more insight into the effectiveness of dyadic planning when it is used to quit an unhealthy behaviour. If cooperating with a non-smoking partner improves the planning intervention, it can be considered as a valuable and low-cost addition to existing smoking cessation interventions.

Partner interactions play an important role in a quit attempt [14, 15, 32]. Therefore, in addition to examining the effectiveness of the planning intervention, we will examine daily partner interactions that might be the working mechanism behind a successful couple intervention. The experience sampling method is an ideal way to examine partner support and its daily effects and fluctuations. An end-of-day diary could show fluctuations in partner support over the course of a quit attempt, the effect that support has on smoking behaviour, and whether couples show differences in support that might be affected by whether their planning was dyadic or individually. For example, it could show differences between the intervention and control group in how the partner responds to difficult moments. If certain partner interactions are found to have a positive influence on the abstinence rate, these could be advised in cessation interventions in which a non-smoking partner is involved. One could instruct partners how to provide support in general and during difficult moments.

As we also measure relationship satisfaction on a daily basis, we are able to see associations between daily interactions in general and related to the quit attempt and how these are associated with relationship satisfaction. Results could show a trade-off; some behaviours might be helpful for the quit attempt, but damaging to the relationship. Therefore, the results of this study can be used not only to provide a better cessation intervention, but also to help maintain a good relationship satisfaction during the difficult time of a quit attempt.

### Strengths and limitations

Strengths of the study include the combination of a promising and low-cost quitting smoking intervention with the experience sampling method. The experience sampling method is the ideal way to examine daily effects and fluctuations in smoking and partner behaviours, which might explain and contribute to the effectiveness of the intervention. Limitations are that we solely rely on self-report and have no no-planning control group. Nevertheless, a meta-analysis showed that self-reports of smoking are accurate in most studies [33]. Ideally, a third group should be added to this study, in which the smoker quits without a planning intervention. However, due to the effect of implementation intentions already found in literature [3, 5], the focus of this study is on examining the effect of the dyadic process. A third group would require a larger sample size, thereby jeopardizing the feasibility of the study.

## Conclusion

This study will provide more insight into whether involving a non-smoking partner could improve planning interventions, and whether and which partner interactions underlie this effectiveness. When proven effective, this planning intervention in combination with coaching for the non-smoking partner will be a valuable and low-cost addition to existing smoking interventions.

## Additional file

Additional file 1: Online informed consent form. (DOCX 102 kb)
