# Supplementary figures and images for: A dyadic planning intervention to quit smoking in single-smoking couples: design of a randomized controlled trial
Source: BMC Psychol. 2018 Nov 12;6:53. doi: 10.1186/s40359-018-0266-8 (PMC6233499; doi:10.1186/s40359-018-0266-8)

**Appendices**

**
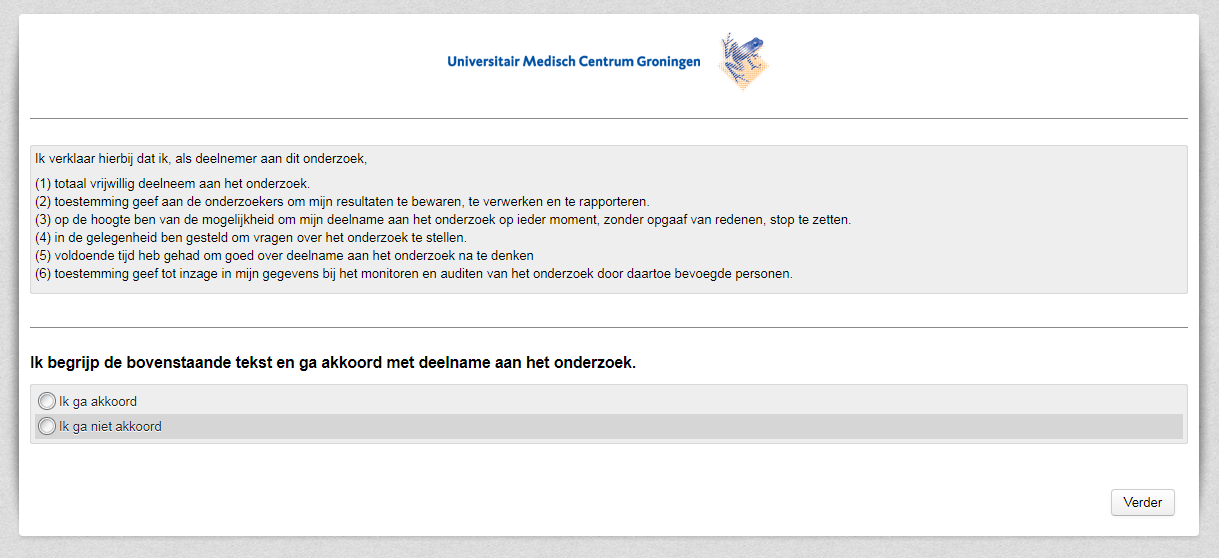
**Screenshot of the online informed consent form.

Supplement: Supplementary file 1 — Online informed consent form. (DOCX 102 kb) [file 40359_2018_266_MOESM1_ESM.docx]
